# Vascular Inflammation and Dysfunction in Lupus-Prone Mice-IL-6 as Mediator of Disease Initiation

**DOI:** 10.3390/ijms22052291

**Published:** 2021-02-25

**Authors:** Paul Marczynski, Myriam Meineck, Ning Xia, Huige Li, Daniel Kraus, Wilfried Roth, Tamara Möckel, Simone Boedecker, Andreas Schwarting, Julia Weinmann-Menke

**Affiliations:** 1 Department of Nephrology and Rheumatology, University Center of Autoimmunity, Johannes-Gutenberg University Mainz, 55131 Mainz, Germany; Paul.Marczynski@unimedizin-mainz.de (P.M.); Myriam.Meineck@unimedizin-mainz.de (M.M.); Daniel.Kraus@unimedizin-mainz.de (D.K.); Tamara.moeckel@unimedizin-mainz.de (T.M.); Simone.Boedecker@unimedizin-mainz.de (S.B.); Andreas.schwarting@unimedizin-mainz.de (A.S.); 2 Institute of Pharmacology, Johannes-Gutenberg University Mainz, 55131 Mainz, Germany; Ning.Xia@unimedizin-mainz.de (N.X.); Huige.Li@unimedizin-mainz.de (H.L.); 3 Institute of Pathology, Medical Center of the Johannes-Gutenberg University Mainz, 55131 Mainz, Germany; Wilfried.Roth@unimedizin-mainz.de

**Keywords:** systemic lupus erythematosus, antibody, cardiovascular disease, macrophage, T Cells, Intima media thickness, cytokines

## Abstract

Background: Systemic lupus erythematosus (SLE) is a chronic inflammatory autoimmune disease and patients are under an increased risk for cardiovascular (CV) events and mortality. The increased CV risk for patients with SLE seems to be caused by a premature and accelerated atherosclerosis, attributable to lupus-specific risk factors (i.e., increased systemic inflammation, altered immune status), apart from traditional CV risk factors. To date, there is no established experimental model to explore the pathogenesis of this increased CV risk in SLE patients. Methods: Here we investigated whether MRL-*Fas^lpr^* mice, which develop an SLE-like phenotype, may serve as a model to study lupus-mediated vascular disease. Therefore, MRL-*Fas^lpr^*, MRL-++, and previously generated *Il6*^−/−^ MRL-*Fas^lpr^* mice were used to evaluate vascular changes and possible mechanisms of vascular dysfunction and damage. Results: Contrary to MRL-++ control mice, lupus-prone MRL-Faslpr mice exhibited a pronounced vascular and perivascular leukocytic infiltration in various organs; expression of pro-inflammatory cytokines in the aorta and kidney was augmented; and intima-media thickness of the aorta was increased. IL-6 deficiency reversed these changes and restored aortic relaxation. Conclusion: Our findings demonstrate that the MRL-*Fas^lpr^* mouse model is an excellent tool to investigate vascular damage in SLE mice. Moreover, IL-6 promotes vascular inflammation and damage and could potentially be a therapeutic target for the treatment of accelerated arteriosclerosis in SLE.

## 1. Introduction

Systemic lupus erythematosus (SLE) is a chronic inflammatory autoimmune disease, affecting predominantly women in childbearing age, and kidney failure is a major cause of morbidity and mortality in these patients [1]. Even if the overall-cause of mortality for SLE decreased over the last decades, partly due to improvements in therapy and diagnosis, patients with SLE show increased overall-cause mortality rates and severely compromised quality of life compared to the general population [2,3]. Main causes of death for patients with SLE are renal and cardiovascular diseases (CVD), and infections [4]. In SLE patients, apart from stroke and end-stage kidney failure, CV mortality is highly increased (up to 3-fold), and incidence of myocardial infarction is 2–10 times higher compared to the general population [3,5,6]. Importantly, in contrast to the decreased SLE-associated mortality over the last decades, mortality due to CVD stayed unchanged and high, with no specific treatment regime available because the underlying pathomechanisms are poorly understood [7]. The increased CV risk for patients with SLE seems to be caused by a premature and accelerated atherosclerosis, attributable to lupus-specific risk factors (i.e., increased systemic inflammation, altered immune status), apart from traditional CV risk factors [8]. The pathogenesis of this premature, accelerated arteriosclerosis is not well defined, with an assumed imbalance between endothelial damage (due to autoantibodies, inflammation, oxidative stress) and protection (capacity of endothelial repair) [9,10]. Once there is vascular damage established, alterations of the microvascular environment produces an acute and chronic immunological response with recruitment of immune cells, such as T cells, monocytes, and macrophages that promote platelet aggregation with microthrombus formation aggravating arteriosclerosis [11,12]. However, the trigger or factors that lead to the accelerated atherosclerosis have not been sufficiently defined and therefore an effective treatment has not yet been developed. 

Thus, an experimental model to investigate molecular mechanisms of the vascular complications is urgently needed, since the treatment of SLE significantly improved but the mortality due to CVD stayed unchanged and high. The continued high incidence of CVD for these patients is particularly relevant to their long-term outcome.

MRL-*Fas^lpr^* mice develop a systemic autoimmune disease akin to human systemic lupus erythematosus and, thus, are a powerful tool to investigate possible pathomechanisms of organ- and tissue-specific manifestations [13,14]. The disease progression in MRL-*Fas^lpr^* mice is rapid, progressive and predictable, but the time frame is sufficiently slow to tease apart the pathogenesis, however the model is sufficiently fast enough to be used in an experimental approach [15]. Not much is known about the vascular damage in this model; in aged MRL-*Fas^lpr^* mice, an impaired endothelium-dependent vasorelaxation was observed [16] and after injection of the TNF-α an increased leukocytes-endothelial interaction in MRL-*Fas^lpr^* mice was detected [17]. Therefore, we propose to test the hypothesis that the MRL-*Fas^lpr^* mouse is an excellent model to identify molecules that mediate accelerated vascular damage in experimental lupus to develop new treatment options and preventive strategies with the long-term goal to translate these findings to human SLE.

## 2. Results

### 2.1. Predominantly Vascular and Perivascular Infiltrates Are Increased in MRL-Fas^lpr^ Mice

**Figure 1:**
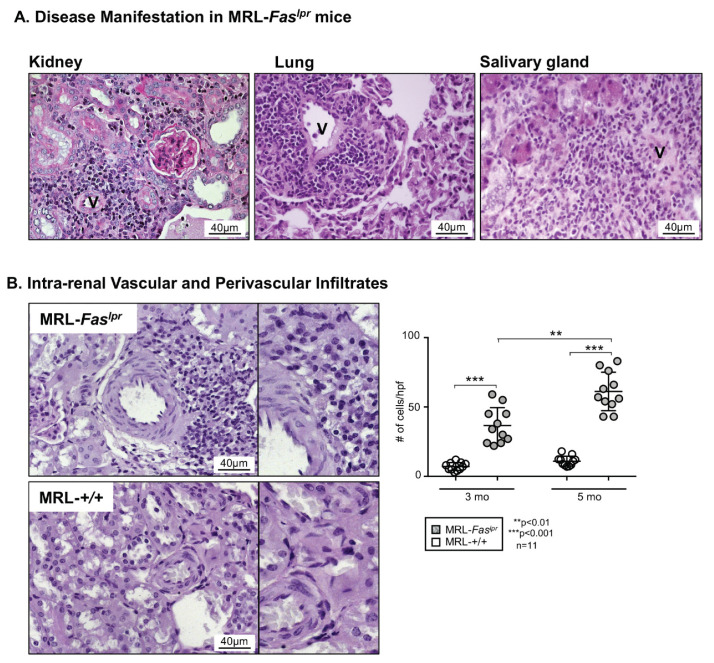
Dominant vascular manifestations on MRL-*Fas^lpr^* lupus mouse model. (**A**) Histopathological imaging of vascular infiltration in the kidney, lung, and salivary glands in 5-month-old MRL-*Fas^lpr^* mice. (**B**) Intra-renal evaluation of vascular and perivascular infiltrates in MRL-*Fas^lpr^* mice (3 and 5 mo of age) compared to MRL-++ mice (control strain), *n* = 11 (a single data refers to a single animal). Representative microphotographs are shown (magnification 40×). Values are means ± SEM. Statistical analysis using Mann–Whitney U Test. v, vascular lumen

To determine the vascular damage in SLE, we examined the vascular and perivascular inflammatory leukocyte infiltrations in various organs in the MRL-*Fas^lpr^* mouse model. We found vascular and perivascular infiltrations of predominantly leukocytes in the kidney, the lung, as well as the salivary glands (Figure 1A). Additionally, although less dominant, there are clear leukocyte infiltrations around glomeruli, bronchioli, and interstitial infiltration in the lung and the kidney (data not shown).

In order to analyze this infiltration in greater detail, we primarily evaluated its occurrence over time with advanced disease manifestations in the MRL-*Fas^lpr^* mouse model. Here, an increase in leukocytes infiltrations was seen in MRL-*Fas^lpr^* mice at 5 mo compared to 3 mo of age (Figure 1B). At the same time, in the MRL-++ control mouse, which shows a slower disease course with first manifestations at 6 months of age, no increased vascular leukocyte infiltration could be detected at 3 or 5 mo of age (Figure 1B).

### 2.2. Increased Vascular and Perivascular Infiltrates of Macrophages and T cells Goes Along with an Increased Intima Media Thickness in MRL-Fas^lpr^ Mice

**Figure 2:**
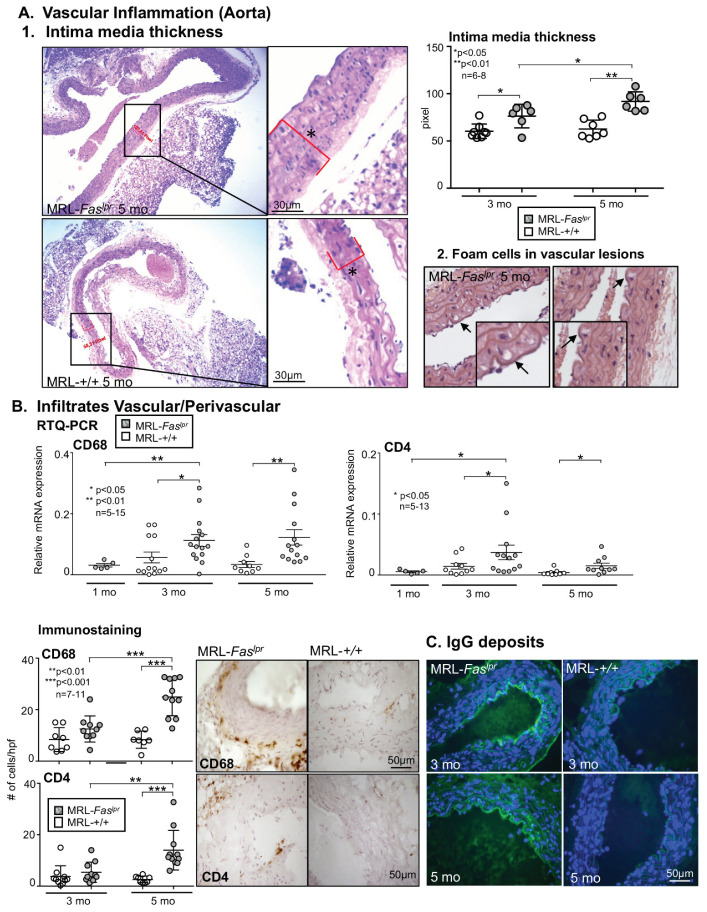
Marked increase in intima–media thickness of aorta and infiltration of vessel wall with macrophages and T cells in MRL-*Fas^lpr^* mice. (**A1**). Intima-media thickness of the aorta in MRL-*Fas^lpr^* compared with MRL-++ mice at 3 and 5 mo of age (*107.81 Pixel and *58.31 Pixel) (**A2**) Foam cells in cell vessel wall of MRL-*Fas^lpr^* mice at 5 mo of age. Representative microphotographs are shown (magnification 20×, enlargement 40×). Arrows denote foam cells. (**B**) Infiltration of the vessel wall with CD68^+^ macrophages and CD4^+^ T cells determined by RTQ-PCR (age 1, 3, and 5 mo of age for MRL-*Fas^lpr^*) as well as by immunostaining (3 and 5 mo of age). Representative microphotographs are shown (magnification 20×, enlargement 40×). hpf, high-power field. (**C**) IgG deposits on the blood vessel wall of MRL-*Fas^lpr^* mice compared to MRL-++ mice at 3 and 5 mo of age. In all experiments *n* = 5–15 mice per group. Values are means ± SEM. Statistical analysis using Mann–Whitney U Test.

In association with increasing vascular and perivascular leukocyte infiltrations in MRL-*Fas^lpr^* mice, there is also a significant increase in intima media thickness evaluated in the aorta in MRL-*Fas^lpr^* mice with advancing disease manifestation. In addition, MRL-*Fas^lpr^* mice show an increased intima media thickness already at 3 mo of age compared with MRL-++ mice of the same age, and this difference becomes more pronounced at 5 mo (Figure 2A1). In addition, we could detect as a typical pathological finding of atherosclerosis early forms of foam cells in the vessel wall of MRL-*Fas^lpr^* mice (Figure 2A2). In addition, we could show in the elastica staining a thinning of the elastic bands and adventitial thickening in MRL-*Fas^lpr^* mice compared with MRL-++ mice at 5 mo of age, typical signs of progressive arteriosclerosis (microphotographs are not shown). We can see in the aorta there is an increase in leukocyte infiltration vascularly and perivascularly. By RTQ-PCR we could detect an increase in CD68^+^ macrophages, as well as CD4^+^ T cells. This infiltration by macrophages and T cells occurs at a very early stage of disease onset between 1 and 3 mo of age in MRL-*Fas^lpr^* mice. In contrast, MRL-++ mice show minimal to no infiltration of macrophages and T cells until 5 mo of age (Figure 2B). Evaluation of the infiltrates by immunostaining showed that the aortic vessel wall infiltrates were dominated by macrophages followed by CD4^+^ T cells in MRL-*Fas^lpr^* mice, corresponding to RTQ-PCR result. Of note, we also detected plasma cells in the vessel wall and the immediate vicinity of the vessel wall in MRL-*Fas^lpr^* mice and mainly no cells in MRL-++ mice (data not shown). Again, there is a clear dependence on advancing disease in the MRL-*Fas^lpr^* mice whereas in the MRL-++ control animals there is little to no infiltration of macrophages and T cells until 5 mo of age. Consistent with the progressive disease activity, we were able to show significant IgG deposits in the vessel wall with increasing age of MRL-*Fas^lpr^* mice compared to MRL-++ mice, comparable to other organs such as the glomeruli in the kidney (Figure 2C).

### 2.3. Inflammatory Markers Like TNF-α Are Not Only Expressed in the Kidney with Advancing Disease, They Are Abundantly Detectable in Aortic Tissue of MRL-Faslpr Mice

**Figure 3:**
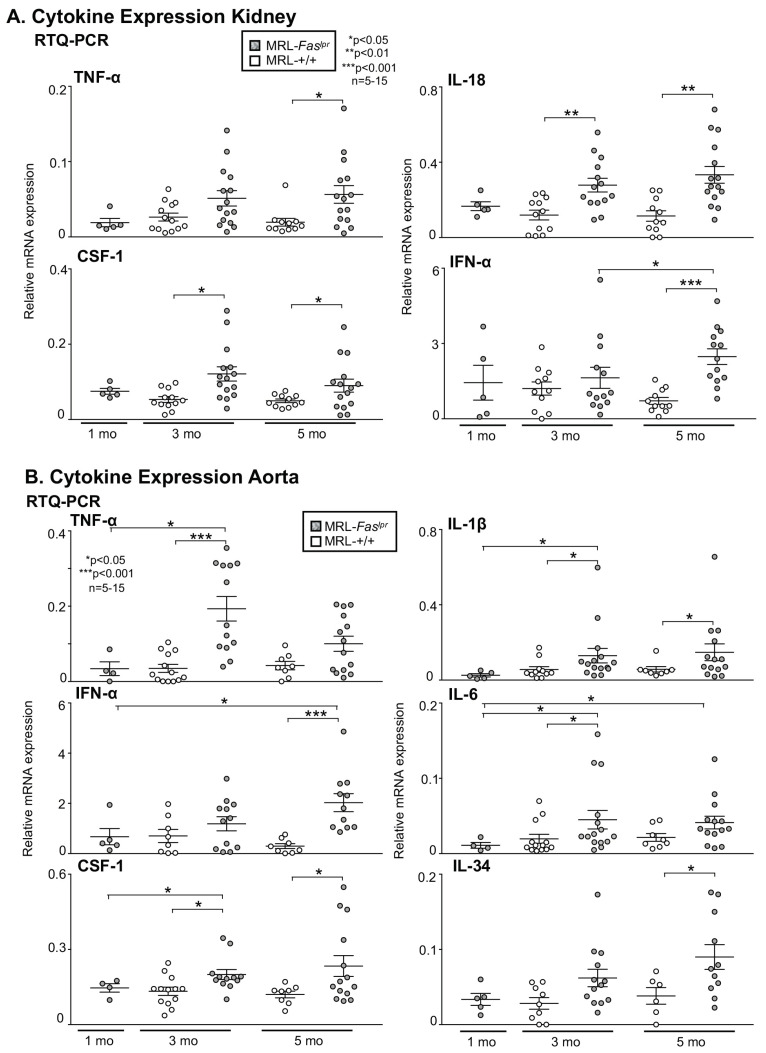
Upregulation of inflammatory cytokines in kidney and aorta with age and in MRL-*Fas^lpr^* compared to MRL-++ mice. (**A**) Expression of TNF-α, IL-18, CSF-1, and IFN-α in the kidney of MRL-*Fas^lpr^* mice compared to MRL-++ mice at 1, 3, and 5 mo of age. (**B**) Expression of IL-6, TNF-α, IL-1β, CSF-1, IL-34 and IFN-α in the aorta of MRL-*Fas^lpr^* mice compared to MRL-++ mice at 1, 3 and 5 mo of age, *n* = 5–15 (a single data point refers to a single animal). Values are means ± SEM. Statistical analysis using Mann–Whitney U Test.

We hypothesized that the rise in vascular and perivascular leukocyte infiltration in the kidney and aorta is accompanied by the expression of proinflammatory cytokines known to promote disease activity in MRL-*Fas^lpr^* mice. In the kidney we could detect an increased expression of TNF-α, IL-18, Colony-stimulating factor-1 (CSF-1) and IFN-α with advancing disease compared to MRL-++ control mice (Figure 3A). To determine whether these cytokines are expressed in the aorta, we analyzed their expression in aortic tissue. In aortic tissue TNF-α, IL-1β, and IL-6 were more robustly expressed compared with MRL-++ mice especially at 3 mo of age (Figure 3B). By comparison, CSF-1, IFN-α, and IL-34 expression was lower. Interestingly, only for IL-6 and IFN-α, there is a continuously rising expression in the aorta with advancing disease from 1 to 5 mo in MRL-*Fas^lpr^* mice (Figure 3B). Therefore, we hypothesized that IL-6, for which a relevant role in the development of lupus nephritis in MRL-*Fas^lpr^* mice has already been shown, might have an important role in vascular injury in the MRL-*Fas^lpr^* mouse model.

### 2.4. Amelioration of Vascular Inflammation in Il6^−/−^ MRL-Faslpr Mice

**Figure 4:**
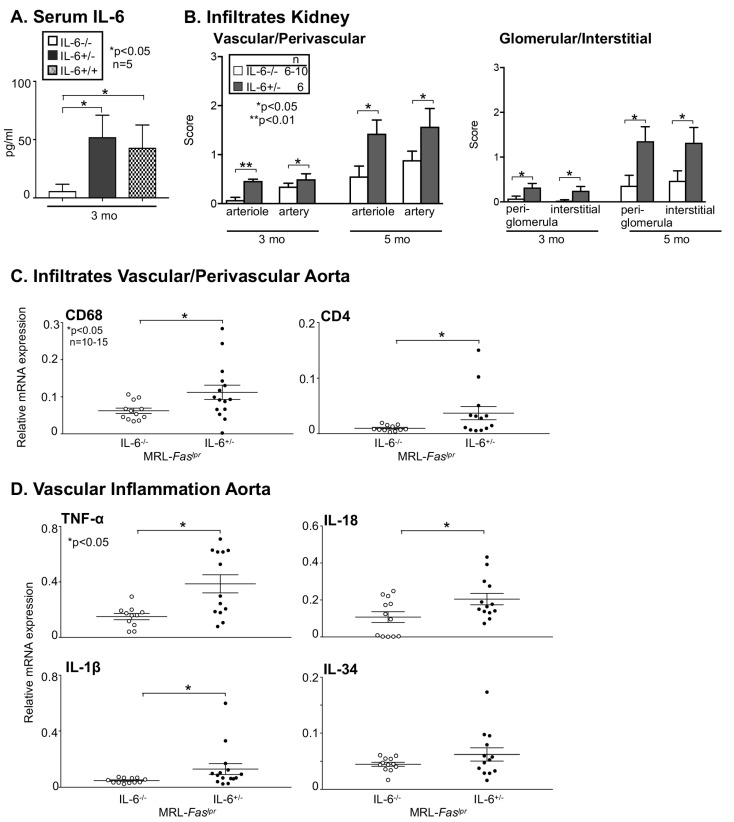
Deficiency of IL-6 results in amelioration of vascular inflammation determined in MRL-*Fas^lpr^* mice. (**A**) Serum IL-6 expression evaluated by ELISA in the serum of *Il6*^−/−^, *Il6*^+/−^ and *Il6*^+/+^ MRL-*Fas^lpr^* mice (*n* = 5 per group). (**B**) Evaluation of intra-renal vascular/perivascular of arterioles and arteries, periglomerular and interstitial in *Il6*^−/−^ MRL-*Fas^lpr^* mice (3 and 5 mo of age) compared MRL-*Fas^lpr^* mice, *n* = 6–10 per group. (**C**) Infiltration of the vessel wall with CD68^+^ macrophages and CD4^+^ T cells determined by RTQ-PCR at 3 mo of age in IL-6^−/−^ MRL-*Fas^lpr^* mice compared to MRL-*Fas^lpr^* mice. (**D**) Expression of TNF-α, IL-1β, IL-18, and IL-34 in the aorta of IL-6^−/−^ MRL-*Fas^lpr^* mice compared to MRL-*Fas^lpr^* to mice at 3 mo of age, *n* = 10–15. A single data point refers to a single animal. Values are means ± SEM. Statistical analysis using Mann–Whitney U Test.

To determine whether IL-6 drives vascular and perivascular infiltration and damage in MRL-*Fas^lpr^* mice, we probed for the age-related increase in intra-renal vascular and perivascular, periglomerular and interstitial infiltrates in *Il6*^−/−^ MRL-*Fas^lpr^* mice compared with *Il6*^+/−^ MRL-*Fas^lpr^* mice (Figure 4A). These examinations were preceded by the evaluation of the comparability of MRL-*Fas^lpr^* mice homozygous and heterozygous for *Il6*. Therefore, we examined IL-6 expression in the serum of these mice. *Il6*^+/−^ and *Il6*^+/+^ showed comparable IL-6 levels in the circulation, whereas IL-6 is undetectable in the *Il6*^−/−^ MRL-*Fas^lpr^* mice (Figure 4A). Intra-renal vascular and perivascular infiltrates as well as periglomerular and interstitial infiltrates are reduced in *Il6*^−/−^ MRL-*Fas^lpr^* mice at 3 and 5 mo of age compared to *Il6*^+/−^ MRL-*Fas^lpr^* mice (Figure 4B). Consistent with the findings in the kidney, the aorta also shows a decreased infiltration of CD68^+^ macrophages and CD4^+^ T cells as determined by RTQ-PCR in *Il6*^−/−^ MRL-*Fas^lpr^* mice 3 mo of age compared to *Il6*^+/−^ MRL-*Fas^lpr^* mice (Figure 4C). Moreover, inflammatory cytokines like TNF-α, IL-18, IL-34, and IL-1β that increase in inflamed aortic tissues in MRL-*Fas^lpr^* mice are decreased in the absence of IL-6 (Figure 4D). Thus, deleting *Il6* suppresses intrarenal vascular and perivascular macrophages and T cell recruitment and infiltration of the aorta by macrophages and T cells resulting in reduced expression of inflammatory cytokines.

### 2.5. IL-6 Mediates Vascular Dysfunction in MRL-Fas^lpr^ Mice

**Figure 5:**
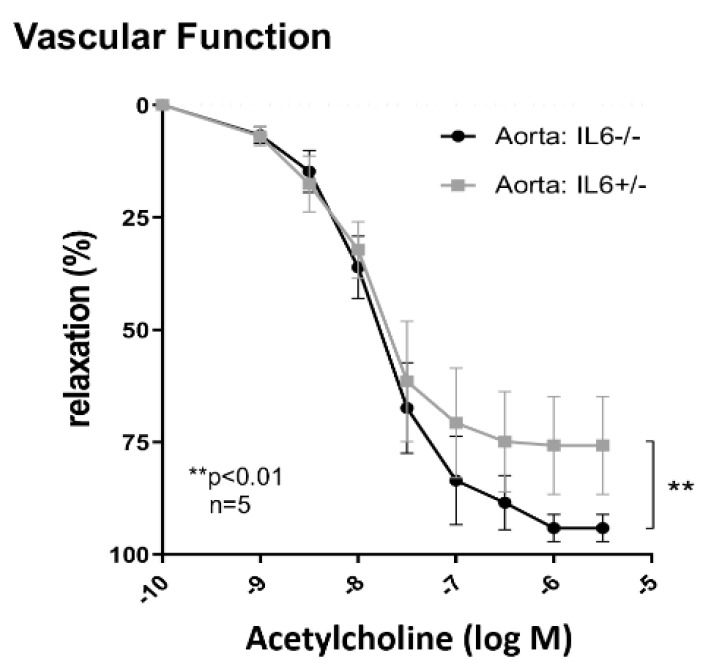
Effect of IL-6 on vascular function in mouse aorta. Thoracic aortic segments were isolated from 3-mo old male *Il6*^+/−^ and *Il6*^−/−^ mice. The rings were contracted with noradrenaline and then relaxed with increasing concentrations of acetylcholine, *n* = 5. Values are means ± SEM. Statistical analysis was performed using two-way ANOVA.

To test the hypothesis that IL-6 promotes vascular inflammation and dysfunction in MRL-*Fas^lpr^* mice, we investigated the vascular function in *Il6*^−/−-^ MRL-*Fas^lpr^* mice compared with *Il6*^+/−^ MRL-*Fas^lpr^* mice at 5 mo of age. *Il6*^−/−^ MRL-*Fas^lpr^* mice showed enhanced vasodilation in response to acetylcholine compared to *Il6*^+/−^ MRL-*Fas^lpr^* mice, indicating an improvement of endothelial function (Figure 5). Collectively, IL-6 induces the expression of cytokines in aortic tissue known to recruit macrophages, and T cells into inflamed aortic tissue and thereby drives vascular inflammation and dysfunction.

In conclusion, our findings demonstrate that the MRL-*Fas^lpr^* mouse model is an excellent tool to investigate the vascular damage, the development of arteriosclerosis and possible therapeutic options in SLE mice.

## 3. Discussion

A growing armamentarium of therapeutics has been instrumental in reducing disease activity and improving the rates of remission of patients with systemic lupus erythematodes. However, despite these advances, cardiovascular mortality remains unacceptably high in SLE [7,18].

Thus, the question arises why patients suffer from accelerated atherosclerosis despite optimized therapy, what the possible pathomechanisms are (immunological, drug-toxic, or SLE-associated) and how we can best investigate them.

Primarily a good experimental model is needed which has a comparable pathogenesis to SLE and is fast enough to achieve efficient research results. The MRL-*Fas^lpr^* mouse is therefore a possible experimental model. We could show that there is a clear vascular involvement with infiltration of macrophages and T cells, that there is expression of proinflammatory cytokines and that this correlates with vascular function.

Most studies report worsening or improvement of endothelium-dependent vasorelaxation in the aorta of lupus-prone mouse strains, e.g., NZB/W or NZM mice [19]. This is based on the considerations that endothelial dysfunction is one of the earliest events in the development of atheroma that promotes disease progression, and triggers CV events [20,21,22]. Moreover, previous studies could show that endothelial progenitor cells (EPC) play a key role in vascular health as the development of endothelial dysfunction in the NZB/W mice correlates with decreased EPC numbers and enhanced EPC death [23,24,25]. In contrast, we now demonstrate that endothelial dysfunction can be detected in MRL-*Fas^lpr^* mice that is not only due to direct alteration of endothelial cell function but is mediated indirectly through perivascular and vascular leukocyte infiltration and proinflammatory cytokine expression in aortic tissue. Furthermore, we demonstrate that the elimination of one cytokine (IL-6) is sufficient to normalize endothelial dysfunction and to significantly reduce perivascular and vascular leukocyte infiltration rates. Thus, this model is ideally suited to differentiate the inflammatory mediated vascular changes in SLE from other causes that may explain the accelerated atherosclerosis, as they reflect the manifestation of human SLE.

In addition, we could demonstrate that not only the endothelial function is impaired, but we were further able to detect a marked increase in intima-media thickness (IMT) of the aorta with advancing disease in MRL-*Fas^lpr^* mice. Intima-media thickness is considered a representative measure of early vascular atherosclerosis—not only in the arteries supplying the brain [26,27]. In epidemiological studies, cardiovascular risk is predicted by IMT. In clinical studies, IMT is often used as a surrogate marker to demonstrate the preventive benefit of antihypertensives or lipid-lowering drugs, for example, as a proxy for clinical events [26,28].

A recent meta-analysis found that therapeutic interventions that had a beneficial effect on IMT progression were also associated with a decrease in cardiovascular events [26]. Thus, with determination of the IMT in mice is an additional reliable tool to evaluate therapy response or effects of preventive measures.

Collectively, to date, there are no other published mouse models that show comparable inflammatory changes in the vasculature and none that are as similar to human SLE in disease manifestations (kidney, skin, salivary glands, joints) as the MRL-*Fas^lpr^* mouse model. Thus, this model is excellently suited to dissect the pathogenesis of accelerated arteriosclerosis in SLE in order to explore new therapeutic targets and strategies.

We could show that with advancing disease activity there is increasing perivascular and vascular infiltration of leukocytes, as well as increased cytokine expression here leading by IL-6 and TNF-α in MRL-Faslpr mice.

Elimination of IL-6 alone results in a much milder vascular manifestation with reduction in infiltrating leukocytes and recovery of endothelial dysfunction. This is in agreement with results showing that IL-6 deficiency was able to reduce kidney pathology and was capable of diminishing lupus activity in MRL-*Fas^lpr^* mice, as well as causing a prolonged survival [29]. It should be noted that next to IL-6 further cytokines such as IFN-γ, IL-10, IL-18, and IL-21 have not negligible roles in the development of autoimmune inflammation in MRL-*Fas^lpr^* mice:

IFN-y receptor-deficient MRL-*Fas^lpr^* mice displayed a reduced severity of nephritis, lymphoid cell proliferation, and, most importantly, the survival of those mice was enhanced compared to IFN-y expressing littermates [30]

While the absence of IL-10 worsens various disease manifestations in MRL-*Fas^lpr^* mice, a blockade of IL-18 receptor signaling delays the onset of disease [31,32]. Moreover, an IL-21-receptor knockout diminishes T and B cell activation and the generation of autoantibodies [33]

Interestingly, we showed that IL-6 deficiency results in an enhanced IL-10 and IFN-γ expression in mice [29] Possibly, IFN-γ has a negative regulatory pathway capable of limiting macrophage mediated renal inflammation. [30]. The influence of the *Il6* knockout on the expression of IL-18 and IL-21 was not tested [29]. Thus, future work on inflammatory processes and their influence on vascular changes in the MRL-*Fas^lpr^* mice will have to deal not only with IL-6 but with the complex network of interacting cytokines in the temporal line of disease development and progression.

Recently it was shown that also in patients with SLE the IL-6 expression correlates with disease activity [34]. Comparatively, therapy with tocilizumab, an anti-human IL-6 receptor antibody, did not show the hoped-for therapeutic success in the case of SLE [35]. While proinflammatory cytokines in general are discussed to influence cardiovascular disease (CVD) in SLE [29] further enhanced IL-6 serum level was associated with coronary artery calcification [36] However, to the best of our knowledge a study evaluating IL-6 as therapeutic option for CVD has not been done yet. 

More research will be needed to understand the complex immune regulation of cytokines by IL-6 and IL-6 trans-signaling in systemic lupus erythematosus and its effects on different organ manifestations. Hence, IL-6 will continue to be in the focus of SLE research and therapy.

In conclusion, we report the novel findings that IL-6 is integral in perpetuating vascular inflammation, dysfunction and damage in MRL-*Fas^lpr^* and that the MRL-*Fas^lpr^* mouse model is an excellent tool to study direct and indirect mechanisms of accelerated arteriosclerosis in SLE.

## 4. Materials and Methods

### 4.1. Mice

MRL-*Fas^lpr^* mice and *Il6* knockout BALB/C mice were purchased from the Jackson Laboratory (Bar Harbor, ME, USA). The mice were kept in the animal facility of the University of Mainz. Only female mice were used for analyses at 1, 3, and 5 mo of age. The use of mice in this study was reviewed and approved by the Standing Committee on Animals at the University of Mainz (23 177-07/G17-1-074, 18-10-17). We generated IL-6-deficient MRL-*Fas^lpr^* mice (*Il6*^−/−^ MRL-*Fas^lpr^*) using a backcross-intercross scheme as described [29]. The progeny were screened by polymerase chain reaction (PCR) amplification of tail genomic DNA using primers for the Il6 wild-type gene (sense, 5′-TTC CAT CCA GTT GCC TTC TTG G -3′; antisense, 5′- TTC TCA TTT CCA CGA CGA TTT CCC -3′) and Il6 deficiency (neomycin resistance insertion; sense, 5′-ATT GAA CAA GAT TTG GGA TTG CAC-3′; antisense, 5′-CGT CCA GAT CAT CCT GAT C-3′). Gel analysis identified the *Il6* and *neoR* gene fragments at 480 and 180 bp, respectively.

### 4.2. Renal Histopathology

Kidneys, lung, aorta, and salivary gland were fixed in 10% neutral buffered formalin, embedded in paraffin, sectioned (4 μm), and stained with periodic acid-Schiff (PAS). Embedding, cutting, and stainings were performed by the Institute of Pathology, Mainz. We scored kidney pathology as previously detailed [8]. Briefly, perivascular infiltrating immune cells in lungs and salivary glands were evaluated as described before [37]. To determine the thickening of the aorta wall, the IMT was calculated at a representative location using AxioVision software (Carl Zeiss Microscopy Deutschland GmbH, Oberkochen, Germany).

### 4.3. Immunostaining

Tissue was embedded in OCT and was snap-frozen in 2-methylbutane with dry ice. We stained cryostat-cut (4 µm) mouse aorta and kidney sections for the presence of: CD4, (553043, BD Pharmingen, San Diego, CA, USA), CD68 (1957, Bio-Rad, formerly Serotec, Hercules, CA, USA), and CD138 (PA5-16918, Invitrogen, Waltham, MA, USA), as previously described [38]. In brief: sections were dried 30 min at RT, then fixed with ethanol (Carl Roth, Karlsruhe, Germany) and acetone (AppliChem, Darmstadt, Germany). Sections were treated with 0.6 % H_2_O_2_ in methanol (or in PBS/0.2% NaN_3_/0.1% BSA for CD4) to block the endogenous peroxidase and subsequently blocked with Avidin/Biotin Blocking Kit (SP-2001, Vector Laboratories Burlingame, CA, USA) and 10% normal rabbit (X0902, DAKO, Glostrup, Denmark) serum in PBS/10% BSA. Incubation with primary antibody was performed O/N at 4 °C. Sections were then incubated with secondary biotinylated Ab (biotinylated rabbit anti rat, BA4001 Vector Laboratories) for 1h at RT. Subsequently sections were incubated with ABC-complex (PK-6100, Vector Laboratories). DAB (SK-4001, Vector Laboratories) was added to stain the cells of interest brown, subsequently sections were counterstained with Mayer’s Hematoxylin (AppliChem). Sections were dehydrated by incubated in 70% EtOH. Followed by 90% EtOH, 100% EtOH then Xylenes before mounting them with Permount Mounting Media (SP15-100, Fisher Chemical, Fair-Lawn, NJ, USA). We enumerated the number of positive cells in 3–10 high-power fields (HPF) depending on the samples size. For the detection of IgG deposits we fixed frozen aorta sections (4 µm) with ethanol (Carl Roth) and acetone (AppliChem), blocked them with normal serum, stained them with DyLight^®^ 488 labeled goat anti-mouse IgG (H+L) (ab96879, Abcam, Cambridge, UK) (used at 1:400 dilution for 1h), mounted them with DAPI-containing mounting medium (H-1200, Vector Laboratories) and analyzed these sections using a fluorescence microscope.

### 4.4. ELISA

IL-6 serum concentrations were determined using the mouse IL-6 DuoSet Kit (DY406, R&D Systems, Minneapolis, MN, USA) according to the manufacturer’s instruction. In brief: Microplates were coated with 2 µg/mL Capture Antibody in PBS O/N at RT. After blocking with 1% BSA in PBS standards, blanks and samples were added, 100 µL, respectively. Murine serum samples were used undiluted. After incubation (O/N, 4 °C) biotinylated Detection Antibody (75 ng/mL) and subsequently Strepatavidin–HRP in blocking buffer was added. Wells were incubated with TMB substrate (555214, BD Biosciences, San Diego, CA, USA) and color development was stopped by adding 1 M H_2_SO_4_. The Absorbance at 450 nm was measured using a microplate reader (ThermoScientific, Waltham, MA, USA).

### 4.5. Quantitative RTQ-PCR

mRNA from shock frozen tissue (aorta and kidney) was isolated with innuPREP RNA Mini Kit 2.0 (Analytik Jena, Jena, Germany) according to the manufacturer’s guideline: 10 mg tissue was lysed in 400 µL RNA Lysis Buffer (Analytik Jena) using the TissueLyser LT (Qiagen, Hilden, Germany) and 5 mm Stainless Steel Beads (Qiagen) for 7.5 min, 50 Hz. DNA was removed by passing the lysates through a DNA binding column (Analytik Jena). Subsequently the lysate was passed through a RNA binding column (Analytik Jena), washed with the kits washing buffers and finally eluted with RNase free water. The RNA concentration was determined using a NanoDrop2000 (ThermoScientific). To quantify *IL-6*, *IL-1β*, *IL-34*, *CSF-1*, *IL-18*, *CD68*, *CD4*, and *TNF-α* expression we performed quantitative RT-PCR using the “QuantiNova SYBR Green PCR Kit” (Qiagen) in combination with the QuantiTect Primer Assays (Qiagen, Mm_IL-6_1_SG, Mm_IL-1β_1_SG, Mm_IL-34_1_SG, Mm_CSF-1_1_SG, Mm_IL-18_1_SG, Mm_CD68_1_SG, Mm_CD4_1_SG, and Mm_TNF-α_1_SG). We used *β-Actin* as a housekeeping gene (Mm_Actb_2_SG) and the following primer from Sigma/Merck (Darmstadt, Germany): *IFN-α* forward: 5′-TGC TGG CTG TGA GGA CAT AC-3′, *IFN-α* reverse: 5′-TCC TCT CCA CAC TTT GTC TCA G-3′. The following real-time PCR protocol was used: 15 min at 95 °C for initial activation of DNA polymerase, 45 cycles of 15 s at 95 °C, 30 s at 57 °C, 30 s at 72 °C, and 30 s at 40 °C for cooling in a Roche LightCycler instrument (Roche Diagnostics, Indianopolis, IN, USA). The data were analyzed by the Δ-CT method.

### 4.6. Assessment of Vascular Function

Thoracic aortas were isolated and dissected into rings of 2–3 mm in length. Isometric tension was recorded using a wire myograph system (Danish Myo Technology, Aarhus, Denmark). The rings were equilibrated for 60 min and contracted two times with 120 mM KCl. For assessment of vascular function, the rings were pre-contracted with noradrenaline to reach the submaximal tension (80% of that obtained with 120 mM KCl), before vasodilatation was induced with acetylcholine [39].

### 4.7. Statistical Analysis

Data represent the means ± SEM prepared using GraphPad Prism software, version 7.0 (Graphpad Software, San Diego, CA, USA). We used the nonparametric Mann–Whitney U test to evaluate *p* values. Comparison of three or more groups were performed using the Kruskal–Wallis test and two-way ANOVA was used to compare the curves.

## Data Availability

The data used to support the findings of this study are available from the corresponding author upon request.

## Abbreviations

Interleukin-6	IL-6
high power field	HPF
Systemic lupus erythematosus	SLE
Antibody	Ab
Cardiovascular disease	CVD

## References

[1] Rees F., Doherty M., Grainge M.J., Lanyon P., Zhang W. (2017). The worldwide incidence and prevalence of systemic lupus erythematosus: A systematic review of epidemiological studies. Rheumatology.

[2] Fors Nieves C.E., Izmirly P.M. (2016). Mortality in Systemic Lupus Erythematosus: An Updated Review. Curr. Rheumatol. Rep..

[3] Thomas G., Mancini J., Jourde-Chiche N., Sarlon G., Amoura Z., Harlé J.R., Jougla E., Chiche L. (2014). Mortality associated with systemic lupus erythematosus in France assessed by multiple-cause-of-death analysis. Arthritis Rheumatol..

[4] Yurkovich M., Vostretsova K., Chen W., Aviña-Zubieta J.A. (2014). Overall and cause-specific mortality in patients with systemic lupus erythematosus: A meta-analysis of observational studies. Arthritis Care Res..

[5] Sharma S.K., Rathi M., Sahoo S., Prakash M., Dhir V., Singh S. (2016). Assessment of premature atherosclerosis in systemic lupus erythematosus patients with and without nephritis. Lupus.

[6] Giannelou M., Mavragani C.P. (2017). Cardiovascular disease in systemic lupus erythematosus: A comprehensive update. J. Autoimmun..

[7] Bakshi J., Segura B.T., Wincup C., Rahman A. (2018). Unmet Needs in the Pathogenesis and Treatment of Systemic Lupus Erythematosus. Clin. Rev. Allergy Immunol..

[8] Menke J., Rabacal W.A., Byrne K.T., Iwata Y., Schwartz M.M., Stanley E.R., Schwarting A., Kelley V.R. (2009). Circulating CSF-1 promotes monocyte and macrophage phenotypes that enhance lupus nephritis. J. Am. Soc. Nephrol..

[9] Liu Y., Kaplan M.J. (2018). Cardiovascular disease in systemic lupus erythematosus: An update. Curr. Opin. Rheumatol..

[10] Skaggs B.J., Hahn B.H., McMahon M. (2012). Accelerated atherosclerosis in patients with SLE--mechanisms and management. Nat. Rev. Rheumatol..

[11] Atehortúa L., Rojas M., Vásquez G.M., Castaño D. (2017). Endothelial Alterations in Systemic Lupus Erythematosus and Rheumatoid Arthritis: Potential Effect of Monocyte Interaction. Mediators Inflamm..

[12] Nhek S., Clancy R., Lee K.A., Allen N.M., Barrett T.J., Marcantoni E., Nwaukoni J., Rasmussen S., Rubin M., Newman J.D. (2017). Activated Platelets Induce Endothelial Cell Activation via an Interleukin-1beta Pathway in Systemic Lupus Erythematosus. Arterioscler. Thromb. Vasc. Biol..

[13] Theofilopoulos A.N., Dixon F.J. (1981). Etiopathogenesis of murine SLE. Immunol. Rev..

[14] Moyer C.F., Strandberg J.D., Reinisch C.L. (1987). Systemic mononuclear-cell vasculitis in MRL/Mp-lpr/lpr mice. A histologic and immunocytochemical analysis. Am. J. Pathol..

[15] Kelley V.E., Roths J.B. (1985). Interaction of mutant lpr gene with background strain influences renal disease. Clin. Immunol. Immunopathol..

[16] Knight J.S., Subramanian V., O’Dell A.A., Yalavarthi S., Zhao W., Smith C.K., Hodgin J.B., Thompson P.R., Kaplan M.J. (2015). Peptidylarginine deiminase inhibition disrupts NET formation and protects against kidney, skin and vascular disease in lupus-prone MRL/lpr mice. Ann. Rheum. Dis..

[17] Marshall D., Dangerfield J.P., Bhatia V.K., Larbi K.Y., Nourshargh S., Haskard D.O. (2003). MRL/lpr lupus-prone mice show exaggerated ICAM-1-dependent leucocyte adhesion and transendothelial migration in response to TNF-alpha. Rheumatology.

[18] McMahon M., Hahn B.H., Skaggs B.J. (2011). Systemic lupus erythematosus and cardiovascular disease: Prediction and potential for therapeutic intervention. Expert Rev. Clin. Immunol..

[19] Thacker S.G., Duquaine D., Park J., Kaplan M.J. (2010). Lupus-prone New Zealand Black/New Zealand White F1 mice display endothelial dysfunction and abnormal phenotype and function of endothelial progenitor cells. Lupus.

[20] Sitia S., Tomasoni L., Atzeni F., Ambrosio G., Cordiano C., Catapano A., Tramontana S., Perticone F., Naccarato P., Camici P. (2010). From endothelial dysfunction to atherosclerosis. Autoimmun. Rev..

[21] Tesauro M., Mauriello A., Rovella V., Annicchiarico-Petruzzelli M., Cardillo C., Melino G., Di Daniele N. (2017). Arterial ageing: From endothelial dysfunction to vascular calcification. J. Intern. Med..

[22] Sciatti E., Cavazzana I., Vizzardi E., Bonadei I., Fredi M., Taraborelli M., Ferizi R., Metra M., Tincani A., Franceschini F. (2019). Systemic Lupus Erythematosus and Endothelial Dysfunction: A Close Relationshi. Curr. Rheumatol. Rev..

[23] Bitterli L., Afan S., Bühler S., DiSanto S., Zwahlen M., Schmidlin K., Yang Z., Baumgartner I., Diehm N., Kalka C. (2016). Endothelial progenitor cells as a biological marker of peripheral artery disease. Vasc. Med..

[24] Altabas V., Altabas K., Kirigin L. (2016). Endothelial progenitor cells (EPCs) in ageing and age-related diseases: How currently available treatment modalities affect EPC biology, atherosclerosis, and cardiovascular outcomes. Mech. Ageing Dev..

[25] Denny M.F., Thacker S., Mehta H., Somers E.C., Dodick T., Barrat F.J., McCune W.J., Kaplan M.J. (2007). Interferon-alpha promotes abnormal vasculogenesis in lupus: A potential pathway for premature atherosclerosis. Blood.

[26] Willeit P., Tschiderer L., Allara E., Reuber K., Seekircher L., Gao L., Liao X., Lonn E., Gerstein H.C., Yusuf S. (2020). Carotid Intima-Media Thickness Progression as Surrogate Marker for Cardiovascular Risk: Meta-Analysis of 119 Clinical Trials Involving 100 667 Patients. Circulation.

[27] Lorenz M.W., Polak J.F., Kavousi M., Mathiesen E.B., Völzke H., Tuomainen T.P., Sander D., Plichart M., Catapano A.L., Robertson C.M. (2012). Carotid intima-media thickness progression to predict cardiovascular events in the general population (the PROG-IMT collaborative project): A meta-analysis of individual participant data. Lancet.

[28] Bots M.L. (2006). Carotid intima-media thickness as a surrogate marker for cardiovascular disease in intervention studies. Curr. Med. Res. Opin..

[29] Cash H., Relle M., Menke J., Brochhausen C., Jones S.A., Topley N., Galle P.R., Schwarting A. (2010). Interleukin 6 (IL-6) deficiency delays lupus nephritis in MRL-Faslpr mice: The IL-6 pathway as a new therapeutic target in treatment of autoimmune kidney disease in systemic lupus erythematosus. J. Rheumatol..

[30] Schwarting A., Wada T., Kinoshita K., Tesch G., Kelley V.R. (1998). IFN-gamma receptor signaling is essential for the initiation, acceleration, and destruction of autoimmune kidney disease in MRL-Fas(lpr) mice. J. Immunol..

[31] Yin Z., Bahtiyar G., Zhang N., Liu L., Zhu P., Robert M.E., McNiff J., Madaio M.P., Craft J. (2002). IL-10 Regulates Murine Lupus. J. Immunol..

[32] Kinoshita K., Yamagata T., Nozaki Y., Sugiyama M., Ikoma S., Funauchi M., Kanamaru A. (2004). Blockade of IL-18 Receptor Signaling Delays the Onset of Autoimmune Disease in MRL-Faslpr Mice. J. Immunol..

[33] Rankin A.L., Guay H., Herber D., Bertino S.A., Duzanski T.A., Carrier Y., Keegan S., Senices M., Stedman N., Ryan M. (2012). IL-21 Receptor Is Required for the Systemic Accumulation of Activated B and T Lymphocytes in MRL/MpJ-Faslpr/lpr/J Mice. J. Immunol..

[34] Ding J., Su S., You T., Xia T., Lin X., Chen Z., Zhang L. (2020). Serum interleukin-6 level is correlated with the disease activity of systemic lupus erythematosus: A meta-analysis. Clinics.

[35] Wallace D.J., Strand V., Merrill J.T., Popa S., Spindler A.J., Eimon A., Petri M., Smolen J.S., Wajdula J., Christensen J. (2017). Efficacy and safety of an interleukin 6 monoclonal antibody for the treatment of systemic lupus erythematosus: A phase II dose-ranging randomised controlled trial. Ann. Rheum. Dis..

[36] Asanuma Y., Chung C.P., Oeser A., Shintani A., Stanley E., Raggi P., Stein C.M. (2006). Increased concentration of proatherogenic inflammatory cytokines in systemic lupus erythematosus: Relationship to cardiovascular risk factors. J. Rheumatol..

[37] Wada Y., Gonzalez-Sanchez H.M., Weinmann-Menke J., Iwata Y., Ajay A.K., Meineck M., Kelley V.R. (2019). IL-34-Dependent Intrarenal and Systemic Mechanisms Promote Lupus Nephritis in MRL-Fas(lpr) Mice. J. Am. Soc. Nephrol..

[38] Iwata Y., Boström E.A., Menke J., Rabacal W.A., Morel L., Wada T., Kelley V.R. (2012). Aberrant Macrophages Mediate Defective Kidney Repair That Triggers Nephritis in Lupus-Susceptible Mice. J. Immunol..

[39] Xia N., Horke S., Habermeier A., Closs E.I., Reifenberg G., Gericke A., Mikhed Y., Münzel T., Daiber A., Förstermann U. (2016). Uncoupling of Endothelial Nitric Oxide Synthase in Perivascular Adipose Tissue of Diet-Induced Obese Mice. Arterioscler. Thromb. Vasc. Biol..

